# The *Aborted Microspores* (*AMS*)-Like Gene Is Required for Anther and Microspore Development in Pepper (*Capsicum annuum* L.)

**DOI:** 10.3390/ijms19051341

**Published:** 2018-05-02

**Authors:** Jinju Guo, Chen Liu, Peng Wang, Qing Cheng, Liang Sun, Wencai Yang, Huolin Shen

**Affiliations:** 1 Beijing Key Laboratory of Growth and Developmental Regulation for Protected Vegetable Crops, Department of Vegetable Science, College of Horticulture, China Agricultural University, Beijing 100193, China; gjj1987.cool@163.com (J.G.); lcis250@163.com (C.L.); JLNWP2017@163.com (P.W.); chengqing2013@126.com (Q.C.); liang_sun@cau.edu.cn (L.S.); yangwencai@cau.edu.cn (W.Y.); 2 Shandong Key Laboratory of Greenhouse Vegetable Biology, Institute of Vegetables and Flowers, Shandong Academy of Agricultural Sciences, Shandong Branch of National Vegetable Improvement Center, Jinan 250100, China

**Keywords:** *Capsicum annuum* L., *CaAMS*, bHLH transcription factor, MYC family, male sterility

## Abstract

Pepper (*Capsicum annuum* L.) is an economically important vegetable crop worldwide. Although many genes associated with anther and pollen development have been identified, little is known about the mechanism of pollen abortion in pepper. Here, we identified and isolated two putative *aborted microspore* (*AMS*) isoforms from pepper flowers: *CaAMS1* and *CaAMS2*. Sequence analysis showed that *CaAMS2* was generated by retention of the fourth intron in *CaAMS1* pre-mRNA. *CaAMS1* encodes a putative protein with a basic helix-loop-helix (bHLH) domain belonging to the MYC subfamily of bHLH transcription factors, and it is localized to the nucleus. Truncated *CaAMS2-1* and *CaAMS2-2* are produced by alternative splicing. Quantitative real-time PCR analysis showed that *CaAMS* (referred to *CaAMS1* and *CaAMS2-2*) was preferentially expressed in stamens and its expression level gradually decreases with flower development. RNA in situ hybridization analysis showed that *CaAMS* is strongly expressed in the tapetum at the tetrad and uninucleate stages. Downregulation of *CaAMS* led to partial shortened filaments, shriveled, indehiscent stamens and abortive pollens in pepper flowers. Several genes involved in pollen exine formation were downregulated in defective *CaAMS*-silenced anthers. Thus, *CaAMS* seems to play an important role in pepper tapetum and pollen development by regulating a complex genetic network.

## 1. Introduction

Anther and pollen development play a crucial role in the life cycle of flowering plants. The number of anther-specific transcripts and sterility-specific transcripts in plants indicate a complex biological process occurring between gametophytic and sporophytic tissues [1,2,3,4,5]. Anthers are surrounded by four distinct somatic layers: the epidermis, endothecium, middle layer, and tapetum, from surface to interior [6]. The tapetum is the innermost layer of the anther wall that surrounds the locule and directly contacts with microspores [6,7]. As a secretory layer, tapetal cells play a crucial role in microspore development by supplying metabolites, nutrients, and sporopollenin precursors [8]. Therefore, the development of tapetal cells differentiation, tapetum development, and subsequent degradation are all closely associated with pollen fertility.

*SPOROCYTELESS* (*SPL*)/*NOZZLE* (*NZZ*) was one of the first genes identified in early anther cell division and differentiation in *Arabidopsis thaliana*. Because *spl/nzz* mutants show normal archesporial cells but defective cell division, these mutants produce aborted microspores and are male sterile [2,9]. *SPL*/*NZZ* regulates early anther cell differentiation by activating downstream genes [10]. Previous research on *A. thaliana* proposed that the leucine-rich repeat receptor-like kinases complex consisting of *EXCESS MICROSPOROCYTES1 (EMS1)/EXTRA SPOROGENOUS CELLS* (*EXS*), *SOMATIC EMBRYOGENESIS RECEPTOR LIKE KINASE 1* and *2* (*SERK1 and SERK2*), and *TAPETUM DETERMINANT 1 (TPD1)* regulate cell-type specification and differentiation. The *EMS1/EXS* gene encodes a leucine-rich repeat receptor protein kinase which putatively localizes to the cell surface and likely plays an essential role in cell-to-cell communication [11,12]. The disruption of *EMS1* leads to the absence of tapetal cells and to the production of sterile pollen grains. Additionally, *serk1 serk2* double mutants are phenotypically similar to *ems1/exs* mutants [13,14]. *TPD1* encodes a small secreted protein that possibly works in coordination with the *EMS1/EXS* gene product to determine tapetal cells fate in *A. thaliana* [15]. A recently proposed model suggested that *TPD1* acts as ligand in its interaction with *EMS1/EXS* and *SERK1/2* to specify tapetal cell fate [4,16].

Other genes involved in late tapetum development or function in *A. thaliana* were also reported. These included *DYSFUNCTIONAL TAPETUM 1 (DYT1)*, *MALE STERILITY 1/2 (MS1/2)*, *ABORTED MICROSPORE (AMS)*, and *AtMYB103*. *DYT1* encodes a putative helix-loop-helix (bHLH) transcription factor and plays a crucial role in tapetal differentiation and early function [17]. The mRNA expression level of *DYT1* in *spl/nzz* and *ems1/exs* mutants suggested that this gene might act on downstream *SPL/NZZ* and *EMS1/EXS* [17]. *AMS* also encodes a bHLH transcription factor and plays a crucial role in tapetum development and post-meiotic microspore development [18]. *MS1* encodes a protein homologous to the plant homeodomain (PHD)-finger family of transcription factors. Its mutation results in premature degeneration of tapetal cells and complete male sterility [19]. The notably reduced expression levels of *MS1* and *AMS* in *dyt1* mutants suggested that both genes act on downstream *DYT1* [17]. The protein encoded by *MS2* shows high homology to a fatty acyl reductase that converts modified lauric acids to sporopollenin precursors, and *ms2* mutations produce nonviable pollen without exine layer [20]. *MYB80* (*MYB103*), which is also required for tapetum and microspore development [21], works downstream of *AMS* and only expresses in the tapetum of developing anthers [22]. The reduced transcript levels of *MS1*, *MS2*, *A6*, and *UNDEAD* in *myb80* mutants suggested these genes work on downstream *MYB80* [23,24].

In the present study, we isolated two *A. thaliana AMS*-like isoforms from pepper (*Capsicum annuum* L.) flower buds, which were designated *CaAMS1* and *CaAMS2*. Two truncated *CaAMS2-1* and *CaAMS2-2* were predicted for fourth intron retention in *CaAMS2* precursor messenger RNA (pre-mRNA). As *CaAMS* is preferentially expressed in stamens, its expression level gradually decreased with the development of the flower buds. RNA in situ hybridization showed that *CaAMS* was strongly expressed in the tapetum at the tetrad and early-mid uninucleate stages. Downregulation of *CaAMS* results in partial shortened filaments, shriveled, indehiscent stamens, and abortive pollens in pepper flowers. Several genes involved in pollen exine formation were significantly downregulated in defective *CaAMS*-silenced anthers. Overall, these traits indicate that *CaAMS* plays an important role in pepper tapetal and pollen development by means of a complex genetic network. Therefore, *CaAMS* seems to be a practical and effective tool to artificially regulate stamens’ fertility and further improve the efficiency of breeding practices.

## 2. Results

### 2.1. Isolation and Sequence Analysis of CaAMS Genes from Pepper

**Figure 1:**
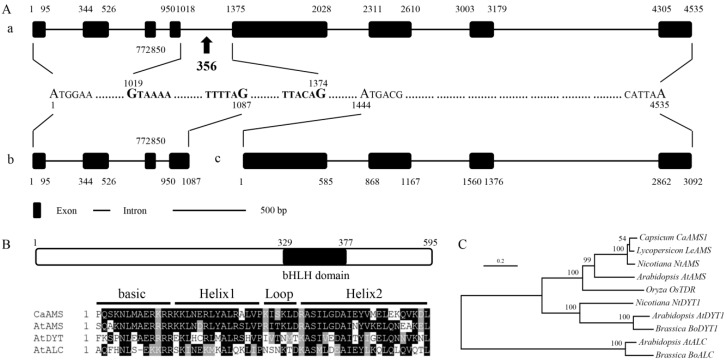
Sequence analysis of *CaAMS*. (**A**) Exon-intron structure of *CaAMSs*. a–c indicate the exon-intron structures of *CaAMS1*, *CaAMS2-1*, and *CaAMS2-2*, respectively. Black boxes indicate exons; Connecting lines indicate introns; Letters in bold indicate the fourth introns inserted; Capital letters indicate the starting and stopping nucleotides. (**B**) Predicted protein structure of *CaAMS1* and sequence alignment of the bHLH domain. The black region indicates the bHLH domain. (**C**) Phylogenetic analysis of the predicted *CaAMS1* proteins with other MYC class bHLH proteins. Amino accession numbers are listed as follows: (1) *Arabidopsis thaliana*: *AtAMS*, AT2G16910.1; *AtDYT1*, AT4G21330; *AtALC*, NP_201512; (2) *Lycopersicum esculentum*: *LeAMS*, XP_019070622; (3) *Nicotiana tabacum*: *NtAMS*, XP_016448229; *NtDYT1*, XP_009773859; (4) *Brassica oleracea*: *BoDYT1*, XP_013669215; *BoALC*, XP_013686240; (5) Oryza sativa: *OsTDR*, Q6YUS3. The bootsrap values from 1000 replicates were indicated on most major nodes.

We isolated two putative *AMS* isoforms from pepper flower buds, designated as *CaAMS1* (Accession No. MH230199) and *CaAMS2*. Through sequence comparison, we found that *CaAMS2* was generated by retention of the fourth intron (Accession No. MH230200) in *CaAMS1* pre-mRNA. The coding sequence of *CaAMS1* contained 1788 bp encoding 595 amino acids, and the predicted molecular weight and isoelectric point of this protein were 68.13 kDa and 5.83, respectively. The exon/intron structure prediction showed that *CaAMS1* has eight exons and seven introns (Figure 1A). The retention-generated *CaAMS2* was 2144 bp with a 356-bp intron insertion between exons 4 and 5 in *CaAMS1*. This alternative splicing probably resulted in a premature translational termination of CaAMS2 at 495 bp (Figure 1A,B) or in an open reading frame (ORF) shift from 852 bp (Figure 1A,C), as predicted using ORF finder. These two truncated *CaAMS2*s were called *CaAMS2-1* (Figure 1B) and *CaAMS2-2* (Figure 1C), respectively. The basic local alignment search tool (BLAST) analysis indicated that the deduced *CaAMS1* was highly similar to the *AMS* homologs found in other species and contained a bHLH domain belonging to the MYC subfamily of bHLH genes (Figure 1B,C). The N-terminus truncated *CaAMS2-2* retained the bHLH domain, while the C-terminus truncated *CaAMS2-1* only retained the N-terminal region characteristic of MYB and MYC transcription factors. Thus, the subsequent analyses mainly focused on *CaAMS1* and *CaAMS2-2*.

### 2.2. Expression Patterns of CaAMS Genes

**Figure 2:**
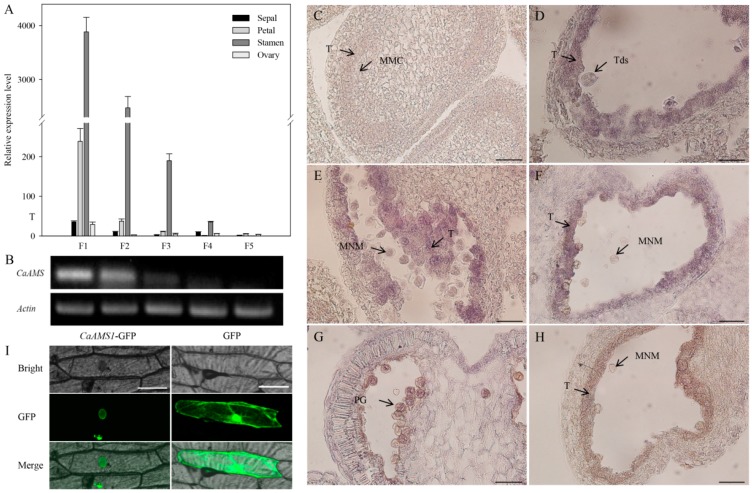
Expression analysis of *CaAMS* in flower buds and subcellular localization of *CaAMS1* protein. (**A**) qRT-PCR analysis of *CaAMS* in flower buds at various developmental stages. F1, F2, F3, F4, and F5 indicate flower buds at tetrad, early-mid uninucleate, late uninucleate, binucleate, and maturing stages, respectively. Data are represented as means ± SD (*n* = 3). (**B**) Semi-quantitative RT-PCR analysis of *CaAMS* in stamens at various developmental stages (same as in qRT-PCR analysis). (**C**–**H**) RNA in situ hybridization with the *CaAMS* probe. (**C**): *CaAMS* signal cannot be detected in tapetum at pollen mother cell stage. (**D**–**F**) Strong signal is detected in the tapetum at the tetrad and uninucleate stages. (**G**) *CaAMS* signal cannot be detected at pollen mature stage. (**H**) The negative control in the uninucleate stage. Only background signal can be detected. T indicates the tapetum; Td indicates the tetrad; MMc indicates the microspore mother cell. MNM indicates the uninucleate microspore. PG indicates the pollen grain. Bar = 200 μm. (**I**) Subcellular localization of *CaAMS* protein in onion epidermal cells. Bars = 100 μm.

The expression patterns of *CaAMS* (except *CaAMS2-1*) in different whorls of floral buds at different developmental stages (i.e., the tetrad, early-mid uninucleate, late uninucleate, binucleate, and maturing stages) were analyzed using quantitative real-time qRT-PCR (Figure 2A). Semi-quantitative RT-PCR analysis was only used for anthers at different developmental stages (Figure 2B). Results showed that *CaAMS* was preferentially expressed in stamens and its expression level gradually decreased with the development of floral buds (Figure 2A,B). Results of RNA in situ hybridization further revealed *CaAMS* signaling cannot be detected in tapetum at pollen mother cell stage (Figure 2C). However, *CaAMS* was strongly expressed in the tapetum at the tetrad and uninucleate stages (Figure 2D–F). Therefore, *CaAMS* might play an important role in pepper tapetum and pollen development.

### 2.3. Subcellular Localization of CaAMS1

After fusing the coding region of *CaAMS1* to the N-terminus of green fluorescence protein (GFP) to produce a CaMV 35S-CaAMS-GFP fusion protein, we performed transient expression assays in epidermal cells of onion to detect the subcellular localization of *CaAMS1*. This result showed that *CaAMS1* is a nuclear localized protein which matched well with the characteristic of transcription factors (Figure 2I).

### 2.4. Virus-Induced Silencing of CaAMS Induces Partial Male Sterility

**Figure 3:**
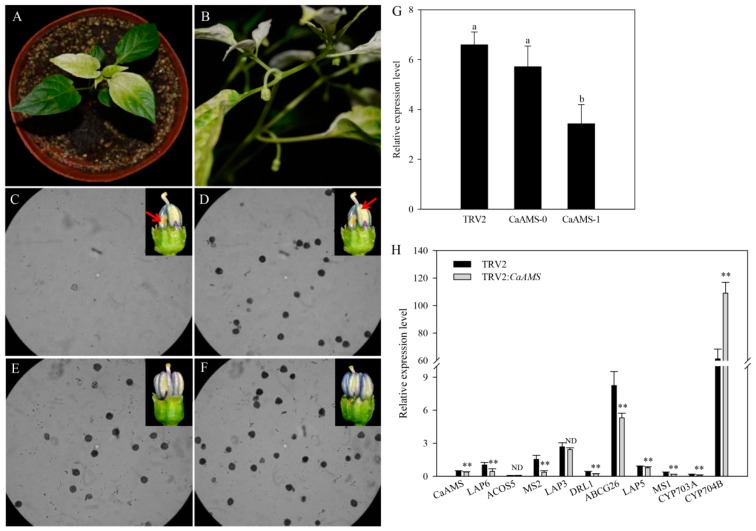
Effects of silencing *CaAMS* in pepper. (**A**,**B**) PDS-silenced plants (negative controls). (**C**–**E**) *CaAMS*-silenced plants. (**C**) Pollen grains in defective stamens of CaAMS-1 type flower buds. (**D**) Pollen grains in normal stamens of *CaAMS*-1 type flower buds. (**E**) Pollen grains in stamens of *CaAMS*-0 type flower buds. (**F**) Pollen grains in TRV2-treated stamens. (**G**) qRT-PCR analysis of *CaAMS* in *CaAMS*-silenced flowers with various phenotypes. Different letters above bars indicate significant differences according to Duncan’s multiple range test (*p* < 0.05). (**H**) Relative expression analysis of genes involved in pollen exine formation by qRT-PCR. *CYP703A*, Cytochrome P450 703A; *CYP704B*, Cytochrome P450 704B; *LAP3/5/6*, Less adhesive pollen 3/5/6; *MS1/2*, Male sterility 1/2; *DRL1*, Dihydroflavonol 4-reductase-like 1; *ABCG26*, ATP-binding cassette transporter G26; *ACOS5*, Acetyl-CoA synthetase 5. ** indicates significant differences at *p* < 0.01, respectively. ND means no difference. Data are represented as means ± SD (*n* = 3).

Virus-induced gene silencing (VIGS) was evaluated by silencing the endogenous tomato PHYTOENE DESATURASE (PDS) gene on TRV2:*PDS*-treated plants. Twenty days after Agrobacterium tumefaciens-infiltration, tender leaves in about 80% (*n* = 10) of the plants appeared photobleached (Figure 3A). Identification based on PCR results indicated that about 65% of the seedlings were successfully transformed. Photobleaching was also detected in flowers (Figure 3B).

There was no obvious phenotype change in the vegetative growth stage. Defective flowers, appearing on the second layer of flowers (40%, *n* = 35), showed partial shortened filaments, shriveled, indehiscent stamens, and abortive pollens (Figure 3C,D). We designated these defective flowers as *CaAMS*-1, and normal flowers as *CaAMS*-0, in TRV2:*CaAMS*-treated flowers. Each defective flower showed one to three degenerated stamens (five stamens per flower) (Figure 3C). The qRT-PCR analysis showed notably lower transcript levels of *CaAMS* in *CaAMS*-1 flower stamens (Figure 3G). The expression levels of *CaAMS* in *CaAMS*-0 flower stamens also decreased, but not differ significantly from that of TRV2-treated flower stamens (Figure 3G). These results further indicated that *CaAMS* might play an essential role in regulating stamen and pollen development in pepper.

### 2.5. Genes Involved in Pollen Formation Have Altered Expression in CaAMS-Silenced Anthers

We performed qRT-PCR using RNA from *CaAMS*-silenced anthers to evaluate if the downregulation of *CaAMS* would affect other genes related to pollen development. Primers for *CaAMS* and *MS1* are listed in Supplementary Table S1 and those for other genes were designed based on our previous work [25]. The expression level of *LAP5/6*, *MS1/2*, *DRL1*, *ABCG26* and *CYP703A* were considerably reduced in *CaAMS*-silenced anthers. This indicated that these genes might work downstream CaAMS and that their normal expression might depend on *CaAMS*. The expression level of *ACOS5* and *LAP3* were slightly altered, and the transcript level of *CYP704B* was notably increased in *CaAMS* silenced anthers, indicating that their expression does not rely on the *CaAMS* gene (Figure 3H).

### 2.6. Promoter Analysis

**Figure 4:**
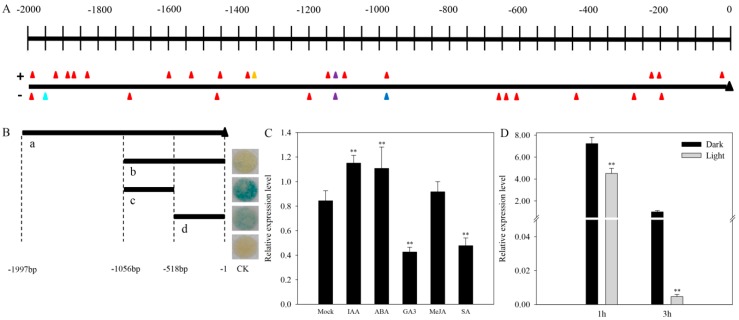
*CaAMS* promoter analysis and expression patterns of *CaAMS* in response to exogenous hormones and light. (**A**) Predicted *cis*-acting elements of the CaSEP5 promoter. Red represents light responsive elements; Blue indicates ABA responsive elements; Purple indicates MeJA responsive elements; Orange indicates SA responsive elements; Aqua indicates GA responsive elements. (**B**) Deletion analysis of the *CaAMS* promoter. Label **a** indicates the *CaAMS* promoter, labels **b**–**d** indicate four truncated promoter fragments and their corresponding GUS expression in tobacco leaf disks. (**C**) Expression patterns of *CaAMS* in response to exogenous hormones. (**D**) Expression patterns of *CaAMS* in response to light. ** indicates significant differences at *p* < 0.01, respectively. ND means no difference. Data are represented as means ± SD (*n* = 3).

A 1997-bp DNA fragment upstream the *CaAMS* start codon was cloned and regarded as the *CaAMS* promoter (Accession No. MH230200). To investigate the regulation mechanisms of *CaAMS*, we analyzed the regulatory elements in the CaAMS promoter region using the PlantCARE database. Twenty-six cis-elements associated with light responsiveness, two ABRE elements involved in abscisic acid (ABA) responsiveness, two methyl jasmonate (MeJA)-responsive motifs (CGTCA/TGACG-motif), one salicylic acid (SA) responsive element (TCA-element) and gibberellin (GA3)-responsive element (GARE-motif) were identified (Figure 4A). Several cis-acting elements involved in defense and stress (e.g., heat, drought) responsiveness were also identified (Supplementary Table S2). The uneven distribution of cis-elements in the *CaAMS* promoter suggested that its transcription is regulated by various environmental signals such as light, hormones, or stress. Promoter deletion analysis indicated that the core region of *CaAMS* is localized at −518 to −1056 bp upstream the ATG translation initiation codon. Inhibitory factors might localize from −1 to −518 bp (Figure 4B).

### 2.7. CaAMS Response to Hormones and Light

*Cis*-element analysis suggested that transcript levels of *CaAMS* might be regulated by various environmental signals, including hormones and light. The expression of *CaAMS* was notably reduced under 100 μmol GA3 and 100 μmol SA treatments, but greatly increased under 100 μmol indole acetic acid (IAA) and 100 μmol ABA treatments; however, *CaAMS* transcript levels showed no response to 100 μmol MeJA (Figure 4C). Light treatment considerably reduced *CaAMS* expression (Figure 4D). These results further illustrated that *CaAMS* transcript levels might be regulated by hormones and light.

## 3. Discussion

### 3.1. Structure and Localization of the CaAMS Gene

MYC class transcription factors are reported to play key roles in cell proliferation, differentiation, and apoptosis [26]. The MYC family proteins consist of three distinct family members, c-MYC, L-MYC, and N-MYC, arising from gene duplication during early evolution [27]. Despite their differences, MYC family members are assumed to work through similar mechanisms [28] and proposed to form homodimers or heterodimers with MYC-associated factor X (MAX) proteins via their helix-loop-helix (HLH) domain [29,30]. The MYC/MAX heterodimers bind variants of the E-box motif “CANNTG”, which can be found in promoters or transcribed sequences of MYC target genes and such binding usually activates the target gene [31,32].

Basic helix-loop-helix proteins are widely distributed in eukaryotic kingdoms. They constitute the largest families of transcription factors and control many biological processes [33,34]. Several MYC class bHLH proteins have been functionally characterized in plants. For example, *delila* (*del*) in *Antirrhinum* sp. regulates the pattern of red anthocyanin pigmentation [35], *alcatraz* (*ALC*) gene in *Arabidopsis* enabling cell separation in fruit dehiscence [36] and *AMS* in *A. thaliana* is involved in the development of tapetal cells and microspores, as well as in filament elongation [18].

In the present study, we isolated and characterized an *A. thaliana AMS* homolog transcription factor from pepper flower buds, named *CaAMS*, which encodes three predicted isoforms generated by alternative splicing. Alternative splicing of pre-mRNA is a regulated process during gene expression that results in multiple proteins encoded by a single gene. Almost all instances of alternative splicing involve one or more of the following basic modules: alternative 5’ or 3’ splice-site choice, cassette-exon inclusion or skipping, and intron retention [37,38]. While *CaAMS1* contains the full-length pre-mRNA, *CaAMS2* retains the fourth intron between exons 4 and 5 (Figure 1A). Intron-containing RNAs are frequently reported to retain in the nucleus, target for degradation, or repress translationally [39]. The *CaAMS2* splice variant was predicted to encode two truncated proteins, designated *CaAMS2-1* and *CaAMS2-2* (Figure 1B,C). Conserved domain prediction indicated that *CaAMS1* contained both the N-terminal region of MYC transcription factors and a bHLH domain, while *CaAMS2-1* encoded a protein that only retained the N-terminal region and *CaAMS2-1* a protein that only retained the bHLH domain. This domain comprises ~60 conserved amino acids and two different functional sub-domains: a DNA binding basic region and two amphipathic α-helices separated by a diverged loop region (i.e., HLH). The N-terminal basic region is involved in DNA binding through the E-box DNA motif “CANNTG”, and the HLH domain promotes the formation of homodimeric or heterodimeric complexes [40,41].

### 3.2. CaAMS1 Is Required for Tapetum and Microspore Development in Pepper

In flowering plants, male sterility depends on the normal development of anthers and microspores. The tapetum is the inner most layer of the anther wall and directly contacts with gametophytes. It is considered to play an essential role in the development of microspores to pollen grains, by supplying nutrients, metabolites, and sporopollenin precursors [42]. Manipulation of crop fertility has significant commercial value for F1 hybrid seed production. Although male-sterile lines have been widely used in pepper breeding, little is known on the mechanism of pollen abortion. Several male-sterile mutants have been associated with tapetum defects [43].

Although the precise mechanisms underlying tapetal development remain highly elusive, several genes involved in tapetum formation, development, and programmed cell death (PCD) have been identified and characterized [44]. In *A. thaliana*, the *AMS* gene encoding a bHLH protein plays a crucial role in the differentiation of tapetal cells and microspores within the developing anther [18]. The predicted *CaAMS1* protein showed the highest similarity to *AMS* homologues in *Solanum lycopersicum*, and only 45% similarity to *A. thaliana AMS*, although all *AMS* homologues contain a bHLH domain belonging to the MYC class of bHLH transcription factors. Nevertheless, *CaAMS1* shares similar exon/intron structures with *A. thaliana AMS* with eight exons and seven introns.

The qRT-PCR analysis indicated that *CaAMS* is preferentially expressed in the stamens of flower buds at the tetrad stage, and that its transcription level gradually decreased as flower buds developed (Figure 2A,B). The RNA in situ hybridization further revealed that *CaAMS* was strongly expressed in the tapetum at the tetrad and the uninucleate stages (Figure 2D–F). However, *CaAMS* is not specific to the tapetum like *AMS* in *Arabidopsis* or *TDR* in rice [5,18]. The *CaAMS* signal was also detected in sepals, petals, and ovaries (Figure 2A). Therefore, the intron-containing alternative splicing might have affected the expression pattern of *CaAMS* in pepper.

Because pepper is highly recalcitrant to in vitro regeneration and genetic transformation, the function of *CaAMS* was evaluated using VIGS. No obvious changes were detected in the phenotype of the vegetative growth stage, but defective flowers appeared on the second layer of flowers. As VIGS is a transient silencing system that cannot completely silent the target genes, the altered phenotypes in *CaAMS*-silenced flowers were usually unstable. Only 40% of the flowers (*n* = 35) were defective, with shortened filaments, shriveled stamens, and abortive pollens. This is consistent with the phenotype of *ams* mutants in *A. thaliana* [18]. Additionally, not all (five) but only one to three stamens degenerated in each defective *CaAMS*-silenced flower. The qRT-PCR analysis showed that *CaAMS* transcript levels were considerably lower in *CaAMS-1* type flower stamens than in normal flower stamens. However, in *CaAMS-0* type flower stamens, the transcript levels of *CaAMS* were not considerably reduced in relation to control/normal flower stamens (Figure 3G). These results indicate that *CaAMS* might play an essential role in regulating stamen and pollen development in pepper.

Previous studies suggested that *AMS* works downstream the *TAPETAL DEVELOPMENT AND FUNCTION 1* (*TDF1*) gene encoding a putative R2R3MYB transcription factor, and that it plays a key role in tapetal differentiation and function [22]. Several tapetum-preferential genes have been identified as involved in the network regulating tapetal cells and microspores development. The *AMS* gene was reported to indirectly induce tapetal PCD or to regulate the downstream pathway of tapetal and pollen formation [44,45]. To test if *CaAMS* downregulation could affect putative pollen formation genes, we performed qRT-PCR using RNA from stamens of defective *CaAMS*-silenced flowers and 10 putative genes involved in pollen exine formation, based on our previous work [25]. Sporopollenin is one of the main components of pollen exine. During the sporopollenin precursor synthetic process, acetyl-CoA released from mitochondria was used as a substrate during fatty acid synthesis (FAS) formation in plastids. After C12, C16 and C18 fatty acids were synthesized, they were modified by Acyl-CoA synthetase5 (ACOS5) and then were transferred to the endoplasmic reticulum (ER). After hydroxylation by CYP703A and CYP704B, the products are CoA-esterified again by ACOS5. Finally, the products were converted to sporopollenin precursors by downstream MS2 and LAP5/6 [25,46,47,48]. ABCG26 was considered to be involved in tapetum-to-microspore sporopollenin monomer transport in *Arabidopsis* [49]. DRL1 and LAP3 were required to synthetic the flavonoids which may serve as sporopollenin precursors [50,51]. Any defects in this process would cause exine formation defect and pollen abortion. In this study, we found that the expression levels of *LAP5/6*, *MS1/2*, *DRL1*, *ABCG26*, and *CYP703A* were considerably reduced in defective *CaAMS*-silenced anthers (Figure 3H), suggesting that the normal expression of these genes might depend on the *CaAMS* gene. Similar mutant phenotypes, mRNA expression levels, and the characteristics of *ACOS5*, *MS2*, and *CYP703A* suggested these might act in a common biochemical pathway [46].

## 4. Materials and Methods

### 4.1. Plant Materials and Growth Conditions

A self-bred pepper (*C. annuum*) line was used in this study. Plants designated for gene cloning, qRT-PCR, and RNA in situ hybridization were grown in experimental fields at China Agricultural University, Beijing, China. For gene cloning and qRT-PCR, flower buds at different development stages (tetrad, early-mid uninucleate, late uninucleate, binucleate, and maturing stages) and dissected floral organs (sepals, petals, stamens, and ovaries) were collected, immediately frozen in liquid nitrogen and stored at −80 °C until use. For VIGS, plant seedlings were grown under 22 °C/16 h day and 18 °C/8 h night conditions in a phytotron.

### 4.2. RNA Extraction and qRT-PCR Analyses

Total RNA was isolated from flower buds using the SV Total RNA Isolation System Kit (Promega Corp., Madison, WI, USA) according to the manufacturer’s instructions. 1 μg total RNA was used to synthesize the first-strand cDNA. Reverse transcription was performed with a PrimeScript™ RT Kit (TaKaRa Bio Inc., Kusatsu, Shiga, Japan). After diluting cDNA products five times, 2 μL aliquots were used for gene cloning and qRT-PCR analysis. The primers used are listed in Supplementary Table S1.

For gene cloning, specific primers were designed based on the pepper genome database (available online: http://peppersequence.genomics.cn/page/species/index.jsp). The amplified cDNA fragments were cloned into pMD 19-T vectors (TaKaRa Bio Inc.), and positive clones were then sequenced at Huada Genetic Sequence Company (Beijing, China).

The qRT-PCR was performed using a GoTaq^®^ qPCR Master Mix (Promega) following the manufacturer’s protocol on an ABI 7500 real-time PCR system (Applied Biosystems, Foster City, CA, USA), under 95 °C for 1 min, followed by 40 cycles of 95 °C for 30 s and 60 °C for 1 min. *Actin* (GQ337966.1) was used as the internal control for its stable expression level in different plant tissues and under variable hormones and abiotic stresses treatments [52]. The qRT-PCR was run with three biological replicates and three technical replicates. Analyses were performed as describes before [25]. The relative expression levels of the target genes were calculated using the 2^−ΔΔ*C*t^ method. The semi-quantitative RT-PCR was performed using the same *CaAMS* primers as the qRT-PCR. Cycling conditions were 3 min at 94 °C followed by 30 cycles of 30 s at 94 °C, 30 s at 54 °C, and 40 s at 72 °C.

### 4.3. Sequence Analysis

Open reading frames were predicted using ORF Finder. The physicochemical characteristics of the proteins were predicted with the ProtParam tool in ExPASy (available online: http://web.expasy.org/protparam/). Deduced amino acid sequences were aligned using Clustal X v.2.0 (available online: http://macdownload.informer.com/clustalx/versions/) and displayed with the BoxShade server v.3.21 (available online: https://embnet.vital-it.ch/software/BOX_form.html). A neighbor-joining (NJ) phylogenetic tree was constructed in MEGA v.5.05 (available online: http://macdownload.informer.com/mega-5/) with 1000 bootstrap replicates.

### 4.4. Promoter Analysis

The 1996-bp fragment upstream the *CaAMS* transcription initiation codon was amplified as the *CaAMS* promoter using specifically-designed primers based on the pepper genome database (available online: http://peppersequence.genomics.cn/page/species/index.jsp). We analyzed the *cis*-elements in the *CaAMS* promoter using PlantCARE database (available online: http://bioinformatics.psb.ugent.be/webtools/plantcare/html/). Serial 5’- and 3’-deletion fragments (A-C) of the full-length promoter were amplified and inserted into the PCAMBIA 1391 vector (without 35S promoter) before the β-glucuronidase (GUS) reporter. The recombined vectors were transformed into the *A. tumefaciens* GV1301 line and then transformed into young tobacco (*Nicotiana benthamiana*) leaves by *A. tumefaciens*-mediated vacuum infiltration. A GUS-staining solution was used as described by Wang et al. [49]. The primer sequences used are listed in Supplementary Table S1.

### 4.5. Subcellular Localization

Full-length *CaAMS* genes (without the terminator codon) were inserted into PUC-SPYNE vectors digested with XbaI and BamH1. Gold particles coated with recombinant plasmids were bombarded onto onion bulb epidermis using a Bio-Rad PDS-1000/He particle delivery system (Bio-Rad Laboratories Ltd., Hercules, CA, USA). The epidermises were cultured in Murashige-Skoog medium for 24 h at 25 °C, in dark. The GFP fluorescence was observed under an Olympus 1X71 confocal microscope (Olympus Corp., Shinjuku, Tokyo, Japan) at 488 nm.

### 4.6. Hormone and Light Treatments

Pepper seedlings at full-blooming stage (i.e., starting to develop the forth layer flower buds) and with uniform growth were selected for treatments. For the exogenous hormone treatments, 10 flower buds at the tetrad stage were randomly collected and embed into gauze soaked with 100 μM hormone solution (IAA, ABA, MeJA, SA, or GA3) in plastic petri dishes for 1 h. For light treatments, five plants were covered with a black cloth for 1 h and 3 h, and five plants were kept under normal lighting conditions (control group). Ten flower buds at the tetrad stage were randomly collected from the five treated plants and immediately frozen in liquid nitrogen. All the experiments were performed in triplicate.

### 4.7. RNA In Situ Hybridization

Fresh shoot apices and flower buds at different developmental stages were collected and fixed in formaldehyde-acetic acid alcohol, dehydrated in graded ethanol series, dewaxed in Histoclear (National Diagnostics, Atlanta, GA, USA), embedded in Paraplast, and sectioned into 10 μm slices. Gene specific probes were generated by RNA polymerase using the DIG RNA labeling kit (Roche, Basel, Switzerland). RNA hybridization and hybridization signaling detection were performed according to Kouchi and Hata [53]. Primers are listed in Supplementary Table S1.

### 4.8. VIGS

The 329-bp C-terminal specific region of *CaAMS* was amplified and inserted into the vector pTRV2 at the BamH1 and Xhol1 sites. The recombined TRV2:*CaAMS* vector was transformed into *A. tumefaciens* GV1301. These transformants and pTRV1 were co-transformed into pepper seedlings with 1–2 euphyllae (three week-old-plants) by *A. tumefaciens*-mediated vacuum infiltration. The TRV2:*PDS* transformants were used as positive controls. Fifteen days after *A. tumefaciens*-infiltration, total RNA was extracted from young leaves to determine the infection efficiency of recombinant TRV in pepper plants. Successfully transformed plants were used for further analysis.

## 5. Conclusions

In conclusion, *CaAMS* is preferentially expressed in the tapetum at the tetrad and the early-mid uninucleate stages. Downregulation of *CaAMS* results in partial shortened filaments, shriveled, indehiscent stamens, and abortive pollens in pepper flowers. Several genes involved in pollen exine formation were downregulated in defective *CaAMS*-silenced anthers. These results indicate that *CaAMS* plays an important role in pepper tapetum and pollen development by regulating a complex genetic network.

## Supplementary Materials

The following are available online at http://www.mdpi.com/1422-0067/19/5/1341/s1. Table S1. Primer sequences used in this study. Table S2. *Cis*-elements in the promoter region of *CaAMS*.

## Abbreviations

bHLH	basic helix-loop-helix
PHD	plant homeodomain
pre-mRNA	precursor messenger RNA
ORF	open reading frame
BLAST	basic local alignment search tool
qRT-PCR	quantitative real-time PCR
VIGS	virus-induced gene silencing
ABA	abscisic acid
MeJA	methyl jasmonate
SA	salicylic acid
GA3	gibberellin
IAA	indole acetic acid
MYC	myelocytomatosis
MAX	MYC-associated factor X
HLH	helix-loop-helix
PCD	programmed cell death
GUS	β-glucuronidase
