# Supplementary material for: The Aborted Microspores (AMS)-Like Gene Is Required for Anther and Microspore Development in Pepper (Capsicum annuum L.)
Source: Int J Mol Sci. 2018 May 2;19(5):1341. doi: 10.3390/ijms19051341 (PMC5983743; doi:10.3390/ijms19051341)
Supplement: Supplementary file 1 [file ijms-19-01341-s001.pdf]

Supplementary

Table S1. Primer sequences used in this study.

Primer Name	Primer Sequence 5'-3'
**Primer sequences for gene cloning**	
CaAMS-F	ATGGAACTCATGCATCTAATGGACA
CaAMS-R	TTAATGATGGTGGTGTCGTTGATAG
**Primer sequences for promoter cloning**	
ProCaAMS-F	CGATCAAACATCCACGTATCAACTG
ProCaAMS-R	GTTTCCTATTAGGGAGAAAAGCAAC
**Primer sequences for RT-PCR**	
qCaAMS-F	CAGATGGGGTTGAAGAGAGC
qCaAMS-R	ATGTAGCGGCAGGGGAGACT
MS1-F	AAGTGCAATCAATGCCATAC
MS1-R	CCCTAATAGCCATCTCAACG
Actin-1-F	ATGGCATCATACTTTCTACAAT
Actin-1-R	CATCAGGTTATCAGTTAGGTCA
Actin-2-F	TCTCAACCCTAAGGCCAACAG
Actin-2-R	CCATCACCAGAGTCCAACACA
**Primer sequences for RNA in situ hybridization**	
CaAMS-Sp6	GATTTAGGTGACACTATAGAATGCTAGTTCTTTGTGAAGGTG TTT
CaAMS-T7	TGTAATACGACTCACTATAGGGGGTGATTATTGTCTAGGTG
**Primer sequences for vector construction**	
**Subcellular localisation**	
CaAMS-GFP-F	TGCTCTAGAATGGAACTCATGCATCTAATGGACA
CaAMS-GFP-R	CGCGGATCCATGATGGTGGTGTCGTTGATAGCGA
**VIGS**	
VCaAMS-F	CGCGGATCCAGTTCTTTGTGAAGGTGTTT
VCaAMS-R	CCGCTCGAGTGGTGATTATTGTCTAGGTG
**Promoter deletion analysis**	
PCaAMS-A-F	CGGGATCCTAACAAACCAAACCCTCATG
PCaAMS-A-R	CCCCCCGGGGTTTCCTATTAGGGAGAAAAGC
PCaAMS-B-F	CGGGATCCTAACAAACCAAACCCTCATGC
PCaAMS-B-R	CCCCCCGGGGAAAAGTGTTTGCCCTCTTCC
PCaAMS-C-F	CGGGATCCCACTTTAGTATTTTGTTGATCTGC
PCaAMS-C-R	CCCCCCGGGGTTTCCTATTAGGGAGAAAAGC

† F= forward, R= reverse; " Red " indicates the restriction enzyme cutting site.
Actin-1 for semiquantitative RT-PCR; Actin-2 for qRT-PCR.

Table S2. Cis-elements in the CaAMS promoter regions.

**Cis-element**	**Function**
3-AF1 binding site	light responsive element
AAGAA-motif	GAAAGAA
ABRE	cis-acting element involved in the abscisic acid responsiveness
AE-box	part of a module for light response
ARE	cis-acting regulatory element essential for the anaerobic induction
AT1-motif	part of a light responsive module
Box 4	part of a conserved DNA module involved in light responsiveness
Box II	part of a light responsive element
Box III	protein binding site
Box-W1	fungal elicitor responsive element
CAAT-box	common cis-acting element in promoter and enhancer regions
CATT-motif	part of a light responsive element
CGTCA-motif	cis-acting regulatory element involved in the MeJA-responsiveness
G-Box	cis-acting regulatory element involved in light responsiveness
GAG-motif	part of a light responsive element
HSE	cis-acting element involved in heat stress responsiveness
MBS	MYB binding site involved in drought-inducibility
RY-element	cis-acting regulatory element involved in seed-specific regulation
Skn-1_motif	cis-acting regulatory element required for endosperm expression
TATA-box	core promoter element around -30 of transcription start
TC-rich repeats	cis-acting element involved in defense and stress responsiveness
TCA-element	cis-acting element involved in salicylic acid responsiveness
TCCC-motif	part of a light responsive element
TGACG-motif	cis-acting regulatory element involved in the MeJA-responsiveness
W box	TTGACC
as-2-box	involved in shoot-specific expression and light responsiveness
ACE	cis-acting element involved in light responsiveness
CAT-box	cis-acting regulatory element related to meristem expression
GARE-motif	gibberellin-responsive element
GATA-motif	part of a light responsive element
I-box	part of a light responsive element
MRE	MYB binding site involved in light responsiveness
OBP-1 site	cis-acting regulatory element
TCT-motif	part of a light responsive element
circadian	cis-acting regulatory element involved in circadian control
